# Real-World Outcomes of Robotic Total Knee Arthroplasty: Five Years’ Experience in a Non-Academic Center

**DOI:** 10.3390/jpm15100482

**Published:** 2025-10-09

**Authors:** Joost Burger, Wei Fan, Sandy Gansiniec, Casper Reinders, Scarlette Kienzle, Clemens Gwinner, Adrianus den Hertog, Arne Kienzle

**Affiliations:** 1 Paracelsus-Klinik Bremen, 28329 Bremen, Germany; joost.burger@pkd.de (J.B.); sandy.gansiniec@pkd.de (S.G.); casper.reinders@pkd.de (C.R.); adrianus.denhertog@pkd.de (A.d.H.); 2 Center for Musculoskeletal Surgery, Clinic for Orthopedics, Charité–Universitätsmedizin Berlin, Corporate Member of Freie Universität Berlin, Humboldt-Universität zu Berlin, and Berlin Institute of Health, 10117 Berlin, Germanyscarlette.kienzle@charite.de (S.K.); clemens.gwinner@charite.de (C.G.); 3 Julius Wolff Institute and Center for Musculoskeletal Surgery, Charité–Universitätsmedizin Berlin, Corporate Member of Freie Universität Berlin, Humboldt-Universität zu Berlin, and Berlin Institute of Health, 13353 Berlin, Germany; 4 Berlin Institute of Health at Charité–Universitätsmedizin Berlin, BIH Biomedical Innovation Academy, BIH Charité Clinician Scientist Program, 10117 Berlin, Germany

**Keywords:** robotic-assisted TKA, real-world data, intraoperative alignment, gap balancing, non-academic hospital

## Abstract

**Background:** Robotic-assisted systems have transformed total knee arthroplasty (TKA), promising improved accuracy and intraoperative consistency, yet real-world data from non-academic centers remain limited. **Objective:** This study evaluates five-year clinical integration of a semi-autonomous, CT-based, robotic-arm-assisted TKA at a tertiary non-teaching hospital in Germany, focusing on planning accuracy, gap balancing, and intraoperative outcomes. **Methods:** We retrospectively analyzed all patients (n = 457) who underwent MAKO-assisted TKA from 2020 to 2025, performed by three orthopedic surgeons using a standardized subvastus approach. We assessed preoperative deformities, intraoperative alignment, implant sizing, and gap balancing. Surgical plans were adapted intraoperatively when indicated. Pre- vs. post-implantation values were compared using slopes to evaluate execution consistency. **Results:** Median patient age was 67.0 years (IQR: 60.0–75.0), with varus in 84.1% (7.0°, IQR: 4.0°–10.0°), valgus in 13.2% (3.0°, IQR: 1.5°–5.8°), and neutral alignment in 2.7%. Flexion contracture occurred in 80.4% (6.0°, IQR: 3.0–10.0%), hyperextension in 12.7% (2.0°, IQR: 1.5°–5.0°). Planning-to-execution consistency was high, even with plan adaptations. Slope values for alignment parameters were: tibial rotation in degrees (slope value: 1.0), femoral sagittal angle in degrees (0.8), tibial sagittal angle in degrees (0.9), coronal posterior condylar angle in degrees (0.9), femoral component size (1.0), tibial component size (1.0). Over 95% of cases showed ≤3.0° deviation between planned and final values. Bone resection concordance showed moderate agreement, with slopes from 0.8 (posterior medial femoral cut in mm) to 0.5 (lateral tibial cut in mm). Gap balancing improved at all stages, with reduced variability in medial/lateral extension and flexion gaps (all *p* < 0.05). Functional reconstruction showed significant improvements in extension, flexion, and deformities (all *p* < 0.001). **Conclusions:** Semi-autonomous, CT-based, robotic-arm-assisted TKA was successfully implemented in this non-academic setting, demonstrating acceptable intraoperative and functional reconstruction outcomes, supporting the feasibility of robotic-assisted surgery outside academic centers.

## 1. Introduction

Robotic-assisted surgical systems have undergone significant technological advancement in orthopedics [1,2]. Particularly in total knee arthroplasty (TKA), the role of robotic assistance has evolved from a supplementary tool to a pivotal technology that enhances surgical precision and enables intraoperative individualization [3]. Compared to traditional TKA, some robotic-assisted systems enable the creation of detailed preoperative plans based on 3D imaging and provide real-time guidance for bone cutting and soft tissue management during surgery [4]. These systems have shown improved intraoperative consistency, optimize postoperative mechanical alignment, and reduce variability in clinical execution, thus minimizing the impact of individual differences [5,6,7]. The semi-autonomous, CT-based, robotic-arm–assisted systems, one of the most widely used types of robotic systems, have demonstrated precise and stable performance in critical aspects of TKA, including bone cutting, alignment angles, prosthesis positioning, and soft tissue gap management. Building on these capabilities, robotic-assisted surgery has the potential to offer advantages over conventional techniques by integrating bone resection with soft-tissue management, thereby supporting the treatment of combined coronal and sagittal deformities [8]. Consequently, it has emerged as a focal point in the clinical advancement of robotic-assisted surgery [9,10].

However, while robotic-assisted systems have demonstrated strong performance in academic centers and controlled, standardized research settings [11], most of these studies have been conducted in large teaching hospitals or multi-center clinical trials, which rely on well-established training systems and technical support platforms [12,13]. In such environments, surgeons typically have access to abundant learning resources and substantial operational experience, allowing them to fully capitalize on the technological advantages of robotic-assisted systems. In contrast, a considerable number of TKA surgeries are performed in non-teaching tertiary hospitals or general medical institutions [14,15]. Although these institutions also possess well-established clinical systems and routine training mechanisms, they may differ from teaching hospitals in terms of resource allocation, surgeon experience, and management strategies for complex cases [16,17]. Interestingly, while one might expect tertiary hospitals to have a more streamlined workflow compared to university hospitals, differences in organization and priorities may nonetheless influence outcomes. Moreover, in real-world settings, clinical environments are often more diverse and complex, with uncertainties in patient demographics, disease conditions, and surgical circumstances that could challenge the overall performance of robotic-assisted systems [18,19].

Consequently, the semi-autonomous, CT-based, robotic-arm–assisted system requires further evaluation through systematic research in these settings to assess its stability and potential for broader implementation in non-academic medical environments.

## 2. Materials and Methods

### 2.1. Research Design

This retrospective study included 457 patients who underwent MAKO-assisted TKA at a non-academic tertiary hospital in Germany between March 2020 and March 2025. The study was approved by the local Ethics Committee (Ärztekammer Bremen, Germany, approval number: 2025-916-retrospektiv).

Inclusion criteria were as follows: (1) a confirmed clinical indication for TKA; and (2) surgery performed with the assistance of the MAKO robotic system (Stryker, Kalamazoo, MI, USA). Patients with both complete and incomplete datasets were included, provided that sufficient data were available for the planned analyses. Exclusion criteria included: (1) scheduled revision TKA; and (2) absence of key preoperative or intraoperative records. All patient data were collected and curated by two trained research assistants from the same orthopedic team [20,21]. The exact number of cases (n) is provided in each corresponding figure legend.

All operations were performed by three experienced orthopedic surgeons using a standardized subvastus approach and the same model of cruciate-retaining (CR) prosthesis (Triathlon^®^, Stryker, Kalamazoo, MI, USA). Preoperative planning was based on high-resolution three-dimensional imaging, which was used to construct a patient-specific skeletal model. Intraoperatively, real-time guidance for bone resection, implant positioning, and soft tissue balancing was provided by the MAKO robotic system.

### 2.2. Data Collection

Baseline information, including age, surgical side, and preoperative deformity status, was collected for all patients.

For each patient, both planned and achieved resection depths were recorded at six anatomical sites: the medial and lateral distal femur, medial and lateral posterior femur, as well as medial and lateral tibia. Alignment parameters collected were femoral and tibial rotation, coronal and sagittal alignment of the femur and tibia, and the posterior condylar axis. Alignment parameters are displayed on the robotic system with respect to the axes (femur and tibia mechanical axes, anteroposterior axes, and mediolateral axes) based on predefined landmarks collected by the Mako Product Specialist and reviewed by the surgeon pre-operatively. The planned and implanted component sizes for the femoral and tibial components were also documented. Agreement between preoperative planning and intraoperative execution, pre- to post-operative changes in resection depths and alignment, and the proportion of cases with resection depth discrepancies ≥ 1 mm and varus/valgus alignment deviations ≥ 3° was statistically evaluated [9,22].

Joint balance was assessed by measuring the medial and lateral gaps between the femur and tibia in both extension and flexion [23,24,25]. Four stages were analyzed: the initial gap based on preoperative CT and reflecting the static bone deformity; the stress gap, recorded intraoperatively under varus-valgus stress to assess soft tissue tension; the correction gap, measured after adjusting component positioning based on the stress test; and the final trial gap, recorded after the insertion of trial components. At each stage, medial and lateral gaps were documented in 10° of flexion to reduce the influence of the posterior capsule and 90° flexion. Changes in gap measurements from the stress to the correction stage were calculated and analyzed for their relationship with the final gaps. The type of alignment used by the surgeons in our series was functional alignment with the goal of achieving medial–lateral gap symmetry in both extension and flexion. While a slightly larger lateral gap in flexion (typically 1–2 mm) was accepted, the overall strategy prioritized balanced gaps across compartments.

Functional improvement and deformity correction were evaluated by comparing preoperative and postoperative measurements. Range of motion, including maximum extension and maximum flexion, was recorded before the bone cuts were performed and after prosthesis implantation. Limb alignment was evaluated in extension, 90° flexion, and full flexion. The robotic system measures limb alignment by calculating the hip–knee–ankle (HKA) angle as the angle between the projections of the femoral and tibial mechanical axes onto the coronal plane. The femoral mechanical axis is defined as the line connecting the hip center, determined as the center of the femoral head, to the femoral knee center, defined as the most distal point of the trochlear groove. The tibial mechanical axis is defined as the line connecting the tibial knee center, corresponding to the exit point of the tibial shaft axis in the coronal and sagittal planes, to the midpoint of the intermalleolar line. To obtain the HKA during flexion, the system projects these axes onto the coronal plane and calculates the resulting angle. It is common for apparent coronal deformity to vary with flexion, and small deviations of 2–3° between extension and flexion are frequently observed and generally accepted [26]. Changes in varus and valgus angles from pre- to post-operative assessments were analyzed.

### 2.3. Statistical Analysis

All statistical analyses were performed using GraphPad Prism version 9.0 (GraphPad Software, San Diego, CA, USA). The Shapiro–Wilk test was used to assess the normality of continuous variables. Data following a normal distribution are presented as mean ± standard deviation (SD), while non-normally distributed data are expressed as median and interquartile range (IQR). Wilcoxon signed-rank tests were used for nonparametric paired comparisons. For time-related variables that did not meet the assumption of normality, the Kruskal–Wallis test was applied, followed by Dunn’s post hoc multiple comparisons. A two-way analysis of variance (ANOVA) was conducted to examine differences in joint gap measurements across intraoperative stages and anatomical positions, with Bonferroni correction used for pairwise comparisons. Correlations between continuous variables were evaluated using linear regression analysis, with regression slopes and corresponding *p* values reported. The variability of balance gaps across different surgical stages was quantified using the coefficient of variation (CV). A *p* value < 0.05 was considered statistically significant.

## 3. Results

### 3.1. Baseline Characteristics

**Figure 1:**
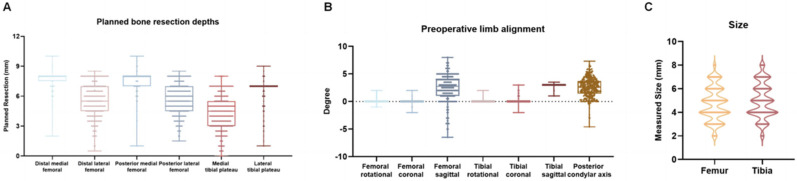
Overview of Preoperative Planning. (**A**) Box and Whisker Plot showing the planned bone resection depths (mm) at the distal and posterior femur (medial and lateral) and the tibial plateau (medial and lateral). (**B**) Box and Whisker Plot showing the preoperative limb alignment (°) in the coronal, sagittal, and rotational planes for both femur and tibia, as well as the posterior condylar axis. (**C**) Violin plot showing planned component sizes for the femur and tibia.

A total of 457 patients were included in the study. The median patient age was 67.1 years (IQR: 60.0–75.0). Preoperative deformity distribution was as follows: 84.1% had varus alignment (median angle: 7.0°, IQR: 4.0°–10.0°), 13.2% had valgus deformity (median angle: 3.0°, IQR: 1.5°–5.8°), and 2.7% exhibited neutral alignment. Flexion contracture was present in 80.4% of patients (median angle: 6.0°, IQR: 3.0°–10.0°); 12.7% presented with hyperextension (median angle: 2.0°, IQR: 1.5°–5.0°); and 6.9% had a neutral extension status. Preoperative planning parameters are summarized in Figure 1.

### 3.2. Consistency of Planning and Execution

**Figure 2:**
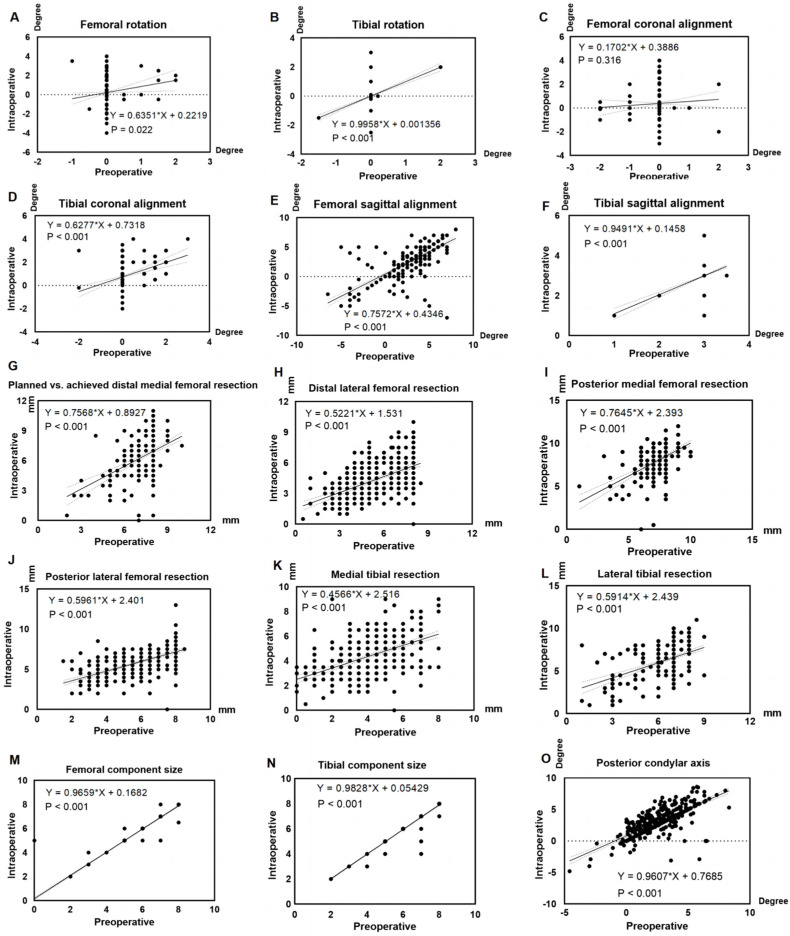
Assessment of consistency between preoperative planning and intraoperative execution. Slope values represent the linear consistency between preoperative planning and intraoperative execution, with a slope of 1 indicating perfect agreement and a slope of 0 indicating no relationship between planned and executed values (**A**) Femoral rotation, (**B**) Tibial rotation, (**C**) Femoral coronal alignment, (**D**) Tibial coronal alignment, (**E**) Femoral sagittal alignment, (**F**) Tibial sagittal alignment, (**G**) Planned vs. achieved distal medial femoral resection, (**H**) Distal lateral femoral resection, (**I**) Posterior medial femoral resection, (**J**) Posterior lateral femoral resection, (**K**) Medial tibial resection, (**L**) Lateral tibial resection, (**M**) Femoral component size, (**N**) Tibial component size, (**O**) Posterior condylar axis (positive = external rotation, negative = internal rotation).

Preoperative planning demonstrated a strong correlation with intraoperative outcomes across multiple alignment parameters. Regression slopes for femoral and tibial rotation were 0.64 (*p* = 0.02) and 1.00 (*p* < 0.001), respectively (Figure 2A,B). For coronal alignment, the slopes were 0.17 (*p* = 0.32) for the femur and 0.63 (*p* < 0.001) for the tibia (Figure 2C,D). Sagittal alignment yielded slopes of 0.76 (*p* < 0.001) for the femur and 0.95 (*p* < 0.001) for the tibia (Figure 2E,F). The posterior condylar axis showed a regression slope of 0.86 (*p* < 0.001; Figure 2O).

Regression slopes for bone resection depth revealed varying degrees of agreement between planned and achieved values. The medial distal and posterior femur showed relatively strong correlations (slopes: 0.76 and 0.77, both *p* < 0.001), while the lateral distal and posterior femur exhibited weaker correlations (slopes: 0.52 and 0.60, both *p* < 0.001; Figure 2G–J). Agreement was lowest for the tibia, with slopes of 0.46 medially (*p* < 0.001) and 0.59 laterally (*p* < 0.001; Figure 2K,L).

Component sizing demonstrated excellent agreement between preoperative planning and intraoperative selection. Regression slopes were 0.97 (*p* < 0.001) for the femoral and 0.98 (*p* < 0.001) for the tibial components. Planned and implanted sizes matched the planned component size in 99.34% of femoral and 99.55% of tibial cases (Figure 2M,N).

The proportion of patients with ≤3° difference between preoperative planning and intraoperative execution was high across all alignment parameters: 98.67% for femoral rotation, 98.45% for femoral coronal alignment, 95.79% for femoral sagittal alignment, 99.79% for tibial rotation, 99.34% for tibial coronal alignment, 100.00% for tibial sagittal alignment, and 97.47% for posterior condylar axis. Table 1 presents the comparative analysis of resection depths and alignment angles between preoperative planning and intraoperative execution.

### 3.3. Gap Adjustment

**Figure 3:**
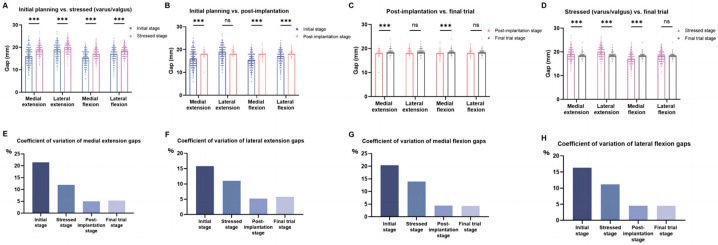
Assessment of joint balance. (**A**) Comparison of gap measurements between the initial planning stage and the gap under varus or valgus stress. (**B**) Comparison between the initial planning stage and the gap after component positioning. (**C**) Comparison between the gap after component positioning and the final trial gap. (**D**) Comparison between the stress stage and the final trial gap. (**E**–**H**) Variability of gap measurements, expressed as the coefficient of variation, shown separately for medial and lateral compartments in extension and flexion. ns, not significant; *** *p* < 0.001.

Significant differences were observed between initial and stress stages for gap measurement points in both extension and flexion, medially and laterally (*p* < 0.001; Figure 3A). Consequently, compared to the initial stage, the medial gaps during extension and flexion, as well as in the lateral gap during flexion were significantly changed after implant position correction (*p* < 0.001; Figure 3B). From the post-implantation to the final trial stage, significant changes were observed in the medial gaps during extension (18.37 ± 0.92 mm vs. 18.64 ± 0.99 mm) and flexion (18.05 ± 0.79 mm vs. 18.50 ± 0.80 mm; *p* = 0.03), while no significant changes were observed for lateral gaps (extension: 18.66 ± 0.97 mm vs. 18.75 ± 1.03 mm; flexion: 18.36 ± 0.83 mm vs. 18.39 ± 0.82 mm; Figure 3C). Comparison between the stress and final trial stages revealed significant differences at all gap measurement points (*p* < 0.05)*,* except for lateral flexion. Specifically, medial gaps decreased from 19.14 ± 2.28 mm to 18.64 ± 0.99 mm in extension and increased from 17.36 ± 2.42 mm to 18.50 ± 0.80 mm in flexion, while lateral gaps showed reductions from 20.07 ± 2.21 mm to 18.75 ± 1.03 mm in extension with no change in flexion (18.39 ± 2.15 mm vs. 18.39 ± 0.82 mm) (Figure 3D). Significant differences were observed between stress to final and post-implantation to final trial measurements at all locations (medial extension: 1.86 ± 1.59 mm vs. 0.95 ± 1.07 mm; lateral extension: 2.15 ± 1.61 mm vs. 0.99 ± 0.88 mm; medial flexion: 2.16 ± 1.72 mm vs. 0.74 ± 0.79 mm; lateral flexion: 1.73 ± 1.41 mm vs. 0.75 ± 0.74 mm; all *p* = 0.02)

CV analysis showed that gap measurements were most variable at the initial stage and became progressively more consistent throughout the procedure. Variability notably decreased after the correction stage, with values stabilizing by the final trial stage (Figure 3E–H).

### 3.4. Postoperative Functional Reconstruction

**Figure 4:**
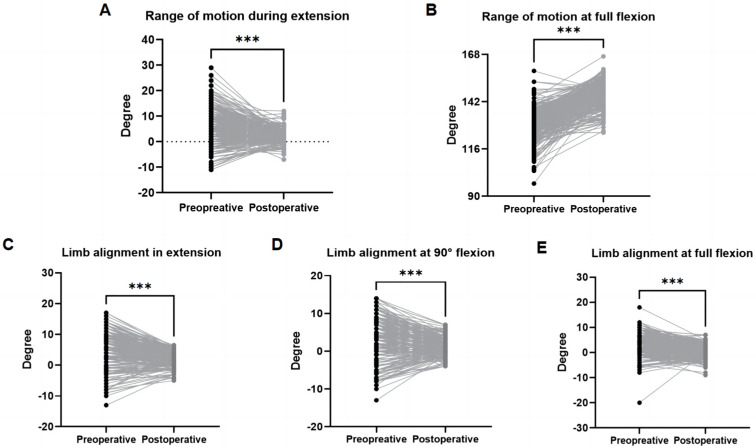
Preoperative and postoperative range of motion and correction of deformity. Positive values indicate flexion or varus alignment; negative values indicate extension or valgus alignment. (**A**) Range of motion during extension represents the maximum extension achieved. (**B**) Range of motion at full flexion represents the maximum flexion achieved. (**C**) Limb alignment in extension. (**D**) Limb alignment at 90° flexion. (**E**) Limb alignment at full flexion *** *p* < 0.001.

Significant postoperative improvements were observed: Both maximum extension and flexion ranges improved significantly (*p* < 0.001; Figure 4A,B) by an average of 4.85° and 18.62°, respectively. Varus/valgus deformity correction was also significant across all evaluated limb positions, including extension (Δ = 4.82°; *p* < 0.001, Figure 4C), 90° flexion (Δ = 3.96°; *p* < 0.001, Figure 4D), and maximum flexion (Δ = 3.58°; *p* < 0.001; Figure 4E).

## 4. Discussion

This study examined the consistency and reliability of robotic-assisted TKA using a semi-autonomous, CT-based, robotic-arm–assisted system across key surgical phases—preoperative planning, intraoperative execution, joint gap balancing, and early postoperative outcomes—based on real-world data from a non-academic tertiary care center. Our findings indicate that the system demonstrates stable performance even in diverse, real-world clinical settings, underscoring its potential for broader implementation across different levels of healthcare institutions.

Intraoperative measurements showed strong alignment with preoperative planning across most key parameters, particularly for rotational alignment and bone resection levels, reflecting the system’s ability to translate planned targets into accurate intraoperative execution. Cadaveric work by Hampp et al. demonstrated lower cutting deviation with robotic assistance compared to conventional methods [27], while Sires et al. reported improved accuracy in prosthesis positioning using the same system as in our study [9]. Implant size predictions were highly consistent with intraoperative component selection, with only minimal deviations observed. Compared to previously reported agreement rates of 86.9% and 93.1% for the tibial component and 84.6 and 96.6% for the femoral component [28,29], our findings indicate substantially higher predictive accuracy. Studies reporting 2D preoperative templating have shown agreement rates of 28.7% for the tibial component and 52.9% for the femoral component [29]. This may support improved preoperative logistics, reduce intraoperative uncertainty, and optimize inventory management for surgical teams with 3D preoperative templating.

In clinical practice, we observed that the robotic-arm–assisted system not only facilitates precise bone resection and alignment planning but also allows for real-time intraoperative adjustment prior to trial implantation. For instance, no significant correlation was found between preoperative and intraoperative values for femoral coronal alignment, suggesting that surgeons frequently modify this parameter intraoperatively based on limb alignment and soft tissue balance rather than strictly adhering to the initial plan. In addition, for femoral coronal alignment, 1.55% had a greater than 3° difference between preoperative planning and intraoperative execution. By contrast, other studies have reported outlier rates in conventional techniques, defined as deviations greater than 3° of the operated plan, of around 16% for femoral coronal alignment [30]. This highlights the robotic system’s dual capacity to execute planned targets accurately while enabling surgical adaptability when warranted. The high resolution of these intraoperative data may also serve as a foundation for future predictive modeling approaches aimed at anticipating surgical adjustments from preoperative and intraoperative parameters to further improve accuracy and clinical outcomes. Deckey et al. reported similar findings, demonstrating that robotic-assisted TKA enables more accurate component positioning and intraoperative balancing compared to conventional techniques [31]. Consistent with this, a recent radiographic study found fewer alignment outliers in robot-assisted procedures across multiple joint angles compared to conventional TKA [32]. However, evidence remains mixed: a recent meta-analysis concluded that robotic assistance in mechanically aligned TKA did not result in superior clinical or radiological outcomes when compared to conventional techniques [33].

In robotic-assisted TKA, flexion and extension gaps are balanced under anesthesia to achieve medial–lateral symmetry rather than replicate weight-bearing conditions. Evidence shows, however, that effective joint gap management is critical for restoring function after TKA [34,35]. In our study, the variability of gap measurements was highest at the beginning of the procedure and steadily declined across subsequent phases, reaching its lowest level at the final trial stage. These findings imply that real-time surgical modifications played a pivotal role in achieving consistent soft tissue equilibrium during the operation. Prior research has similarly demonstrated the potential of robotic assistance to enhance intraoperative balancing. Gazgalis et al. reported improved compartment balancing in flexion with robotic-assisted TKA compared to conventional methods, although no differences were observed in mid-flexion or extension [36]. Selvanathan et al. concluded that robotic systems improve the precision of bone resections and allow for more targeted soft tissue releases to optimize balance [37]. Additionally, Yu et al. found that ligament balancing in robotic-assisted TKA may offer advantages in soft tissue preservation and bone conservation, though clinical and radiographic outcomes at two-year follow-up remained comparable to conventional techniques [6]. More importantly, Graichen et al. demonstrated that the surgeon’s choice of bony alignment influences gap balancing, meaning that alignment strategy modifies the patient-specific phenotype and consequently affects ligament tensioning [38]. Together with our results, these findings underscore the role of robotic platforms in facilitating more controlled and adaptable intraoperative joint balancing strategies that reflect the alignment strategy selected by the surgeon.

Postoperative functional outcome in our cohort was characterized by marked improvements in range of motion. Although this study did not systematically explore the causal relationship between intraoperative gap management and postoperative outcomes, previous research has shown that asymmetrical gaps may lead to instability, pain, or restricted motion [39], while implant malposition is a known risk factor for aseptic loosening [40,41]. These findings indicate that modern TKA robotic systems’ capability for precise intraoperative gap management could foster improved biomechanical conditions, thereby supporting better postoperative outcomes. Supporting this, Valtanen et al. demonstrated that dynamic, force-controlled gap balancing during TKA leads to improved Knee Society Scores, highlighting the clinical benefits of refined soft tissue control in robotic workflows [42]. Further evidence from a recent cohort study showed that tibial polyethylene insert thickness—a surrogate marker for surgical accuracy—was more reproducible in robotic-assisted procedures than in navigation-assisted or manually performed TKA, with a relatively short learning curve required to achieve this consistency [43]. Additionally, robot-assisted techniques have been associated with improved compartment balancing, particularly in flexion, though not uniformly across all movement phases [36]. Collectively, the evidence supports a role for robotic assistance in improving intraoperative balancing, which could translate into more reliable surgical outcomes.

Real-world data captures the variability inherent in routine surgical practice, including differences in patient characteristics and surgeon experience that are often minimized in controlled clinical trials. Within this heterogeneous environment, the robotic-arm-assisted system used in our study demonstrated consistent performance, supporting its technical reliability and adaptability across diverse clinical conditions. Nonetheless, several limitations should be noted. The lack of a control group and single-center design limits direct comparisons with conventional TKA. Although all eligible patients were included, it remains possible that more complex cases are preferentially referred to academic centers, potentially limiting generalizability. Furthermore, long-term functional outcomes and postoperative complications were not assessed. Broader adoption of robotic-assisted knee arthroplasty may benefit from standardized outcome tracking across different healthcare settings.

## 5. Conclusions

In real-world, non-academic clinical settings, semi-autonomous, CT-based, robotic-arm-assisted TKA demonstrated close agreement between preoperative planning and intraoperative execution. It also enabled precise joint gap control across surgical phases. These intraoperative outcomes were accompanied by improvements in range of motion and coronal limb alignment. The findings support the clinical applicability of robotic-assisted TKA in non-academic environments. Wider implementation efforts may benefit from coordinated outcome tracking and extended follow-up across diverse care settings.

## Figures and Tables

**Table 1:** Comparative analysis of preoperative and intraoperative execution measurements for resection depths and alignment angles.

Parameter	Preoperative Median (IQR)	Intraoperative Median (IQR)	Δ Median (IQR)	*p* Value
Femoral rotation (°)	0.0 (0.0–0.0)	0.0 (0.0–1.0)	0.5 (0.0–1.5)	<0.01
Femoral coronal alignment (°)	0.0 (0.0–0.0)	0.0 (0.0–1.0)	0.0 (0.0–1.0)	<0.01
Femoral sagittal alignment (°)	3.0 (1.5–4.0)	2.5 (1.0–4.0)	0.0 (0.0–1.0)	<0.01
Tibial rotation (°)	0.0 (0.0–0.0)	0.0 (0.0–0.0)	0.0 (0.0–0.0)	0.05
Tibial coronal alignment (°)	0.0 (0.0–0.0)	0.5 (0.0–2.0)	0.5 (0.0–1.5)	<0.01
Tibial sagittal alignment (°)	3.0 (3.0–3.0)	3.0 (3.0–3.0)	0.0 (0.0–0.0)	0.39
Posterior condylar axis (°)	2.6 (1.5–3.7)	3.0 (1.8–4.3)	0.5 (0.0–1.4)	<0.01
Distal medial femoral resection (mm)	8.0 (7.5–8.0)	7.0 (5.5–8.0)	1.0 (0.5–2.0)	<0.01
Distal lateral femoral resection (mm)	5.5 (4.5–7.0)	4.0 (3.5–5.5)	1.0 (0.5–2.0)	<0.01
Posterior medial femoral resection (mm)	8.0 (7.0–8.0)	8.5 (7.5–9.0)	1.0 (0.0–1.5)	<0.01
Posterior lateral femoral resection (mm)	5.5 (4.5–6.5)	6.0 (5.5–6.5)	0.5 (0.0–1.0)	0.02
Medial tibial resection (mm)	4.5 (3.0–5.5)	4.5 (3.5–5.5)	1.0 (0.5–1.5)	0.05
Lateral tibial resection (mm)	7.0 (7.0–7.0)	6.5 (5.0–7.0)	1.0 (0.0–1.9)	<0.01

IQR, interquartile range (25th–75th percentiles); Δ, difference between intraoperative and preoperative values.

## Data Availability

The raw data supporting the conclusions of this article will be made available by the authors on request.
